# Uncovering mechanisms of hyperexcitability in tauopathies with functional genomics

**DOI:** 10.1002/alz.083573

**Published:** 2025-01-03

**Authors:** Steven C Boggess, Vaidehi Gandhi, Ming‐Chi Tsai, Yu‐Ying Chou, Jesse E. Hanson, Martin Kampmann

**Affiliations:** ^1^ University of California, San Francisco, San Francisco, CA USA; ^2^ Genentech, South San Francisco, CA USA; ^3^ Memory and Aging Center, UCSF Weill Institute for Neurosciences, University of California, San Francisco, San Francisco, CA USA; ^4^ Chan Zuckerberg Biohub, San Francisco, CA USA; ^5^ University of California San Francisco, San Francisco, CA USA

## Abstract

**Background:**

Patients with Alzheimer’s Disease and related dementias associated with the accumulation of pathological tau (tauopathies) in neurons have an increased incidence of epileptic episodes and sub‐clinical epileptiform activity. This neuronal hyperexcitability represents some of the earliest changes in patient brains, is associated with more severe symptoms, and presents an opportunity for early therapeutic intervention. Despite these provocative observations, the molecular details of how tau and neuronal excitability are connected in tauopathies remain unknown. Thus, identification of how pathological tau influences neuronal activity will reveal novel neuronal biology, further elucidate the in vivo function of tau, and possibly reveal new therapeutic strategies and targets. We are developing methods for identifying modifiers of neuronal hyperexcitability in tauopathies through functional genomics.

**Methods:**

The calcium integrator CaMPARI2 was incorporated in iPSCs that also constitutively express catalytically dead Cas9 (dCas9) fused to a KRAB transcriptional repressor to inhibit expression of a gene targeted by a sgRNA (CRISPRi). We demonstrated that photoconversion of CaMPARI2 is dependent on neuronal activity, and that it reports the observed differences between neurons carrying a disease‐causing mutation in tau and a wild‐type isogenic control. iPSCs are differentiated into neurons by overexpression of NGN2 and activity was recorded via CaMPARI2 photoconversion after 3 weeks of maturation. We performed CRISPRi screening on more that 3000 genes, including genes associated with neurodegenerative disease. FACS was used to sort these neurons into two populations: one consisting of the highly active (high CaMPARI2 ratio) neurons and one consisting of sparsely active (low CaMPARI2 ratio) neurons. Frequency of each sgRNA was identified by next‐generation sequencing.

**Result:**

We have identified genetic modifiers that affect neuronal excitability specifically in WT or tau‐mutant neurons, but not the other. Validation of hits by whole‐cell electrophysiology recordings indicates that the CaMPARI screen can detect a diversity of mechanisms that impact neuronal excitability. Gene Ontology enrichments implicate mitophagy and metabolism as potential drivers of changes to neuronal excitability.

**Conclusion:**

Functional genomics screens are being developed to identify modifiers of excitability in disease and healthy neurons. Further screening and validation experiments will reveal mechanisms that lead to hyperexcitability in tauopathies and potential targets for therapeutic intervention.

